# The miR-146a SNP Rs2910164 and miR-155 SNP rs767649 Are Risk Factors for Non-Small Cell Lung Cancer in the Iranian Population

**DOI:** 10.1155/2020/8179415

**Published:** 2020-11-20

**Authors:** Neda K. Dezfuli, Ian M. Adcock, Shamila D. Alipoor, Sharareh Seyfi, Babak Salimi, Maryam Mafi Golchin, Neda Dalil Roofchayee, Mohammad Varhram, Esmaeil Mortaz

**Affiliations:** ^1^Department of Immunology, School of Medicine, Shahid Beheshti University of Medical Sciences, Tehran, Iran; ^2^Department of Immunology, School of Medicine, Dezful University of Medical Sciences, Dezful, Iran; ^3^Airways Disease Section, National and Lung Institute, Imperial College London, Dovehouse Street, London, UK; ^4^Priority Research Centre for Healthy Lungs, Hunter Medical Research Institute, The University of Newcastle, Newcastle, New South Wales, Australia; ^5^Molecular Medicine Department, Institute of Medical Biotechnology, National Institute of Genetic Engineering and Biotechnology, Tehran, Iran; ^6^Chronic Respiratory Diseases Research Center, National Research Institute of Tuberculosis and Lung Diseases (NRITLD), Shahid Beheshti University of Medical Sciences, Tehran, Iran; ^7^Department of Biotechnology, Ferdowsi University, Mashhad, Iran; ^8^Mycobacteriology Research Center, National Research Institute of Tuberculosis and Lung Diseases (NRITLD), Masih Daneshvari Hospital, Shahid Beheshti University of Medical Sciences, Tehran, Iran; ^9^Clinical Tuberculosis and Epidemiology Research Center, National Research Institute of Tuberculosis and Lung Diseases (NRITLD), Shahid Beheshti University of Medical Sciences, Shahid Beheshti University of Medical Sciences, Tehran, Iran

## Abstract

**Background:**

Lung cancer is one of the leading causes of death worldwide. MicroRNAs (miRNAs) are small noncoding RNAs that regulate gene expression and may act as both tumor suppressors and as oncogenes. The presence of single nucleotide polymorphisms (SNPs) inside the miRNA genomic region could affect target miRNA maturation, expression, and binding to its target mRNA and contribute to cancer development. Previous studies on the SNPs Rs2910164 in miR-146a and Rs767649 in miR-155 showed association with non-small cell lung cancer (NSCLC) development. Thus, the aim of this study was to detect any correlation between those SNPs in Iranian NSCLC patients.

**Methods:**

In a small cohort study, 165 NSCLC patients and 147 noncancer controls were enrolled between Apr 2015 and Sep 2019 at the Masih Daneshvari Hospital, Tehran, Iran. Allele frequencies from the genomic DNA of blood cells were studied using PCR-RFLP and their association with the risk of lung cancer was evaluated.

**Results:**

The rs2910164C allele (OR = 1.56, 95% CI = 1.10–2.21, *p* = 0.012) and CC genotype (OR = 2.93, 95% CI = 1.07–7.9, *p* = 0.034, respectively) were associated with a significantly increased risk for lung cancer compared to that for the GG genotype. When patients were stratified according to smoking exposure, no association with rs2910164 variants was found. The AT genotype (OR = 0.57, 95% CI = 0.33–0.99, *p* = 0.048) and the A allele frequency (OR = 0.58, 95% CI = 0.35–0.98, *p* = 0.043) in rs767649 were lower in NSCLC patients in comparison with the control group. In addition, the rs767649 AT genotype frequency in smoking controls was higher than in smoking NSCLC patients (OR = 0.44, 95% CI = 0.21–0.90, *p* = 0.024). No association was found between rs2910164 and rs767649 variants and stage or type of NSCLC.

**Conclusion:**

Our finding suggests that miR-146a rs2910164 and miR-155 rs767649 polymorphisms may be considered as genetic risk factors for the susceptibility to NSCLC in the Iranian population. However, a larger multicenter study across Iran is needed to confirm these findings.

## 1. Introduction

Lung cancer is the most common cancer worldwide and is associated with high mortality rates [1]. The two main subtypes of lung cancer are small-cell (SCLC) and non-small-cell lung carcinoma (NSCLC). NSCLC encompasses over 80–85% of lung cancers although SCLC, which accounts for 12% of all cases, is more aggressive than NSCLC [1]. In spite of the considerable improvement in diagnosis and treatment, lung cancer remains the leading cause of cancer-related deaths globally [2]. Improving our knowledge of the molecular pathology of NSCLC is important for ensuring earlier diagnosis and successful treatment. Although smoking is one of the major risk factors for lung cancer, nonsmokers also suffer from the disease [3]. Indeed, lung cancer is now considered as a multifactorial disease resulting from a combination of genetic background and lifestyle habits such as diet, smoking, and environmental pollution [4].

Previous studies have demonstrated the importance of multiple genes in lung cancer pathology including P53, Myc, and BRCA1 [1]. In addition to these genes, microRNAs (miRNAs) have recently been identified as important factors in the development of numerous cancers [5]. miRNAs are a family of endogenous small, noncoding RNAs that regulate gene expression posttranscriptionally. miRNAs are implicated in the regulation of almost all biological processes and may function as either oncogenes or tumor suppressor elements. Dysregulation of miRNA expression is reported in a wide array of cancers where they play key roles in oncogenesis including the promotion of invasion, metastasis, and angiogenesis [5].

Recently, attention has been focused on the effects of single nucleotide polymorphisms (SNPs) within the noncoding regions of miRNAs. SNPs can significantly affect the production or processing of miRNAs by modulating their expression or by altering their association with the 3′ untranslated regions (UTRs) of their target mRNAs. Thus, SNP-induced alterations in miRNA maturation, structure, and expression may impact on cancer susceptibility [6]. Rs2910164 and rs767649 polymorphisms have been frequently observed in association with lung [7, 8], breast [9], cervical [10], and hepatocellular [11] cancers. In the current study, we aimed to assess the possible association between miR-146a rs2910164 and miR-155 rs767649 polymorphisms in an Iranian cohort with newly diagnosed NSCLC.

## 2. Material and Methods

### 2.1. Patients

One hundred and sixty-five patients with newly diagnosed NSCLC before the onset of treatment with drugs or radiation therapy and with no history of other cancers or inflammatory diseases (aged 58.5 ± 8.6 years) were recruited at the Masih Daneshvari Hospital, Tehran, Iran, between Apr 2015 and Sep 2019. One hundred and forty-seven age- and gender-matched controls that had a general health check-up with a negative history of cancer and inflammatory diseases were also enrolled. Demographic information of the study participants is shown in Table 1. The Ethics Committee of the Masih Daneshvari Hospital approved the study and all subjects gave their written informed consent (Ethics committee approval number: IR.SBMU.MSP.REC.1397.525).

### 2.2. Genotyping

Genomic DNA was isolated from peripheral blood cells (200 *μ*l whole blood) using a DNA extraction kit (High Pure PCR Template Preparation Kit, Roche, Germany, Cat.No.11796828001) according to manufacturer instructions. The DNA concentration was measured by Nanodrop 2000 (Thermo Fisher, USA). Specific SNPs were genotyped using polymerase chain reaction–restriction fragment length polymorphism (PCR-RFLP) with the PCR reaction performed using super PCR master mix (YEKTA TAJHIZ AZMA, Tehran-Iran, Cat NO: YT1553-YT1554) using a Thermal Cycler instrument (Bio-Rad, CA, USA).

The primer sequences for each PCR reaction is shown in Table 2. The cycle parameters for the PCR analysis were as follows: initial denaturation at 95°C for 5 min, 35 cycles of denaturation at 94°C for 30 sec, annealing at 58°C for 1 min, extension at 72°C for 1 min, and a final extension at 72°C for 10 min. To identify the miR-146 C/G polymorphism, the PCR product was digested with the restriction enzyme mnlI (Thermo Fisher, USA, REF: ER1071) by incubating the samples at 37°C for 4 h. The miR-155 T/A polymorphism PCR product was incubated at 37°C overnight with the restriction enzyme TSP45I (Thermo Fisher, USA, REF: ER1511) and the digestion products were detected by 3% agarose gel electrophoresis.

### 2.3. Statistical Analysis

The differences in genotype distribution for the two analyzed SNPs between NSCLC and the healthy subjects were analyzed using the Chi-square test. Deviations of the genotype frequencies in the controls from those expected under the Hardy–Weinberg equilibrium (HWE) were assessed by a goodness-of-fit *χ*^2^ test. All statistical tests were carried out using SPSS-25 software (SPSS, Inc.) and *p* values ≤ 0.05 were considered statistically significant.

## 3. Results

The study included 165 NSCLC cases and 147 healthy controls (Table 1) with a mean age of 58.5 and 52.6 years in cases and controls, respectively. The age and gender distributions were similar between the two groups (*p* > 0.05). The distribution of the rs2910164 and rs767649 genotypes in the control group by HWE was (*X*^2^ = 1.57, *p* value = 0.209 and *X*^2^ = 1.85, *p* value = 0.173, respectively), which indicates the randomness of the control samples.

**Figure 1:**
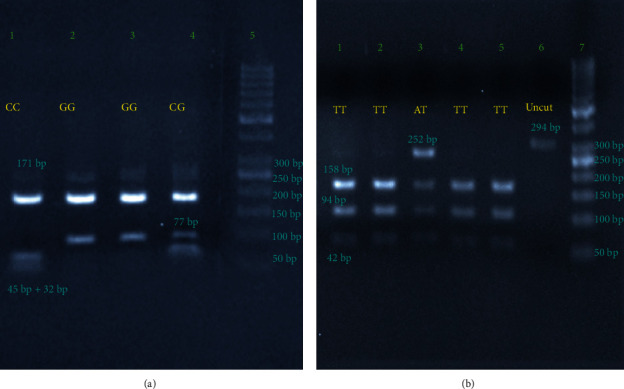
(a) The miR-146a PCR product and the genotypes. Lane 1 shows the CC genotype (bands: 171 bp and 45 + 32 bp); lanes 2 and 3 show the GG genotype (band at 171 and 77 bp); lane 4 shows the CG genotype (bands: 171, 77, and 45 + 32 bp); and lane 5 is the DNA ladder with specific size markers labeled. (b) The miR-155 PCR product and its genotypes. Lanes 1, 2, 4, and 5 show the TT genotype (bands: 158,94 and 42 bp); lane 3 shows the AT genotype (bands at 252, 158, 94, 42 bp); lane 6 shows uncut PCR product at 294 bp; and lane 7 is the DNA ladder.

For rs2910164, the uncut PCR product size was 248 bp and the digested products in patients with the G allele show bands at 77 and 171 bp and for patients with the C allele the bands were at 171, 45, and 32 bp products (Figure 1(a)). The PCR product size for rs767649 was 294 bp and the digested products in subjects with the T allele were 158, 94, and 42 bp and for those with the A allele were 252 and 42 bp (Figure 1(b)).

Genotypes and allele frequency for rs2910164 and rs767649 are shown in Table 3. Statistical analysis using the Chi-square test demonstrated that the C allele and the CC genotype of miR-146a rs2910164 was correlated with NSCLC (OR = 1.56, 95% CI = 1.10–2.21, *p* = 0.012; OR = 2.93, 95% CI = 1.07–7.9, *p* = 0.034, respectively) compared to the GG genotype.

We also show that TT is a common genotype and that AA is a rare genotype of miR-155 rs767649 in Iranian NSCLC patients and control subjects. The AT genotype and the A allele frequency in miR-155 rs767649 variants are lower in NSCLC patients in comparison with the control group (OR = 0.57, 95% CI = 0.33–0.99, *p* = 0.048; OR = 0.58, 95% CI = 0.35–0.98, *p* = 0.043). Moreover, the A allele has an inverse association with lung cancer (Table 3).

rs2910164 variants were not associated with smoking status (Table 4), whereas the AT genotype frequency in smoker controls was higher than in smoking NSCLC patients with the rs767649 variant (OR = 0.44, 95%CI = 0.21–0.90, *p* = 0.024) (Table 4). However, no evidence of association was found between rs2910164 and rs767649 polymorphisms and the stage or the type of NSCLC (*p* > 0.05, Table 5). The distribution of rs2910164 alleles and rs767649 alleles is summarized in Table 6.

## 4. Discussion

In the current study, we found that the miR-146a rs2910164 and miR-155 rs767649 variants were significantly associated with the risk of NSCLC in an Iranian population. In particular, we demonstrate a significantly increased risk of NSCLC in subjects with the CC genotype of miR-146a rs2910164, compared to subjects with the GG genotype. In contrast, the AT genotype of miR-155 rs767649 is protective against NSCLC. There was no link between miR-146a polymorphisms and smoking status although the AT genotype frequency of miR-155 was lower in smokers with NSCLC than in noncancerous smoking controls. No polymorphisms were associated with NSCLC stage or type.

EGFR expression and NF-*κ*B pathway activation have important roles in lung cancer progression [12, 13] and miR-146a and miR-155 have important roles in these pathways, and as their functions complement each other we decided to carry out a study on them [14, 15]. Because of the limitation in this study, we selected only the most common SNPs based on a previous study [7, 16, 17]. The evidence from previous studies suggests that the SNPs studied here were the most important in the regulation of miR-146a and miR-155, and lung cancer in other populations.

miR-146a negatively regulates severe inflammation [18] in an NF-*κ*B-dependent manner and can bind to sequences within the 3′-UTRs of the TNF receptor-associated factor 6 (TRAF 6) and IL-1 receptor-associated kinase 1 (IRAC1) genes [18]. NSCLC is associated with enhanced inflammation [19] and changes in miR-146a expression may enhance the risk of lung cancer. miR-146a has been reported to be both up- and downregulated in cancer, and its role varies according to the type of the tumor [14]. miR-146a expression is significantly lower in the lung cancer tissue compared with the “normal” tissue from control subjects and is considered to act as a tumor suppressor by targeting epidermal growth factor receptor (EGFR) expression. EGFR has an important role in lung cancer [14] and EGFR-tyrosine kinase inhibitors (EGFR-TKIs) are used successfully as targeted therapies [20]. miR-146a induces cell cycle arrest at the G0/G1 phase and thereby suppresses the proliferation of cancer cells in the lung [21] and increases cisplatin sensitivity by targeting JNK2 and cyclin *J* [22, 23].

In previous studies, Jia and colleagues showed that the miR-146a CC genotype and C allele distribution in NSCLC patients were significantly higher (*p* = 0.03 and 0.03, respectively) and concluded that the rs2910164 polymorphism of miR-146a was associated with an increased risk of NSCLC in a Chinese population [8]. A meta-analysis has also reported that the rs2910164 polymorphism is associated with both NSCLC and SCLC [16]. In addition, Jeon and colleagues showed that the miR-146a CG or GG genotypes decrease the risk for lung cancer, compared to the CC genotype in a Korean population [24]. The same study also demonstrated that miR-146a expression levels in tumor tissues from never-smokers were significantly higher in those patients with a GG genotype than in those with a CC or CG genotype [24]. One study on Chinese nonsmoking females showed that individuals with the CG genotype of miR-146a rs2910164 had less risk of lung cancer than those carrying the CC genotype [25].

miR-146a polymorphisms have also been linked to a number of other cancers [9, 26–29]. A recent meta-analysis showed that the CC genotype of miR-146a rs2910164 was associated with susceptibility to NSCLC but not to hepatocellular carcinoma and gastric cancer [17]. In stratified analysis by cancer type and ethnicity, it was reported that these polymorphisms were associated with a significant risk of lung cancer, breast cancer, and colorectal cancer in Asian but not Caucasian populations [30].

miR-155 expression is significantly upregulated in lung cancer tissues, plasma, and sputum, and is associated with the risk of NSCLC [7], possibly through a modulatory effect on NF-*κ*B activity [7, 10]. miR-155 expression is upregulated in lung cancer and could be considered as a potential diagnostic marker as it indicates a poor prognosis of the disease [31]. One meta-analysis study showed that miR-155 could be a potential biomarker for lung cancer detection but not an effective biomarker for the prediction of lung cancer outcomes [32].

miR-155 levels are negatively correlated with the levels of SOCS1, SOCS6, and PTEN [33]. SOCS1 induces apoptosis and inhibits the growth of cancer cells, whereas SOCS6 is associated with disease recurrence. Importantly, PTEN is a tumor suppressor in NSCLC [7, 33]. In the A549 lung cancer cell line, miR-155 modulates cellular apoptosis and DNA damage through an Apaf-1-mediated pathway [34]. miR-155 overexpression also increases cell proliferation, migration, and invasion by oxidative stress pathways. Interestingly, the T allele is associated with reduced sensitivity to chemotherapy and radiotherapy, and its presence indicates a poor disease prognosis [7]. The T allele increased the transcriptional activity of miR-155, and patients with the rs767649-TT genotype had the highest risk ratio for NSCLC and had a reduced response to radiotherapy or chemotherapy [7], which is the opposite to the protective effect of this allele in cervical cancer [10].

In conclusion, the current study indicates that the miR-146a rs2910164 and miR-155 rs767649 polymorphisms are associated with the risk of NSCLC, with the AT genotype of miR-155 rs767649 being protective against NSCLC. These polymorphisms can regulate miRNA expression and affect downstream signaling pathway, which is associated with cancer susceptibility, and may be useful in diagnostic and therapeutic purposes. However, larger sample sizes with diverse ethnicities are needed to extend the findings.

## Figures and Tables

**Table 1:** Distribution of selected demographic variables of lung cancer and control subjects.

Factors	Lung cancer, *n* = 165 (%)	Control, *n* = 147 (%)	*p* value
*Age (years, mean* *±* *SD)*	58.5 ± 8.6	52.6 ± 8.3	

*Gender (n, %)*			
Male	128 (77.58)	115 (78.23)	0.32
Female	37 (22.42)	32 (21.77)	

*Smoking status (n, %)*			
History	102 (61.82)	93 (63.27)	0.44
No history	63 (38.18)	54 (36.73)	

*Histological subtype (n, %)*			
ADC	135 (81.81)		
LCC	6 (3.65)		
SCC	24 (14.54)		

*Stage (n, %)*			
I	4 (2.42)		
II	18 (10.91)		
III	33 (20.00)		
IV	110 (66.67)		

**Table 2:** PCR primer sequences used and expected fragment sizes.

Polymorphism	Primer sequence	Restriction enzyme	Product size (bp)
Rs2910164	F: 5′-AGAACTGAATTCCATGGGTTG-3′ R: 5′-TGCTTAGCATAGAATTCAAGTC-3′	mnlI	Uncut product: 248G Allele: 171 + 77 C allele: 171 + 45 + 32
Rs767649	F: 5′-CCT GTA TGA CAA GGT TGT GTT TG-3′ R: 5′-GCT GGC ATA CTA TTC TAC CCA TAA-3′	TSP451	Uncut product: 294 A allele: 252 + 42 T allele: 158 + 94 + 42

**Table 3:** Genotypic and allelic frequencies of miR-146a rs2910164 and miR-155 rs767649 polymorphisms in non-small-cell lung cancer (NSCLC) and control subjects.

Polymorphism	Lung cancer, *n* = 165 subjects (%)	Control, *n* = 147subjects (%)	OR (95% CI)	*p* value

*miR-146a Rs2910164*				
*Allele*				
G	219 (66.36%)	222 (75.55%)	1 (reference)	
C	111 (33.64%)	72 (24.45%)	1.56 (1.10–2.21)	0.012
*Co-dominant*				
GG	69 (41.8%)	81 (55.1%)	1 (reference)	
GC	81 (49.1%)	60 (40.8%)	1.58 (0.99–2.51)	0.051
CC	15 (9.1%)	6 (4.1%)	2.93 (1.07–7.9)	0.034
*Dominant*				
GG	69 (41.8%)	81 (55.1%)	1 (reference)	
GC + CC	96 (58.2%)	66 (44.9%)	1.7 (1.09–2.67)	0.019
*Recessive*				
GG + GC	150 (90.9%)	141 (95.9%)	1 (reference)	
CC	15 (9.1%)	6 (4.1%)	2.35 (0.88–6.22)	0.085

*miR-155 Rs767649*				
*Allele*				
T	290 (87.87%)	242 (82.3%)	1 (reference)	
A	40 (12.13%)	52 (17.6%)	0.64 (0.41–1.00)	0.051
*Co-dominant*				
TT	131 (79.3%)	102 (69.3%)	1 (reference)	
AT	28 (16.9%)	38 (25.8%)	0.57 (0.33–0.99)	0.048
AA	6 (3.6%)	7 (4.7%)	0.66 (0.21–2.04)	0.47
*Dominant*				
TT	131 (79.3%)	102 (69.3%)	1 (reference)	
AA + AT	34 (20.6%)	45 (30.6%)	0.58 (0.35–0.98)	0.043
*Recessive*				
AT + TT	159 (96.3%)	140 (95.2%)	1 (reference)	
AA	6 (3.6%)	7 (4.7%)	0.75 (0.24–2.29)	0.62

OR = odds ratio.

**Table 4:** The association between SNPs and the risk of NSCLC stratified by smoking.

SNP	Genotype	Nonsmokers (*n* = 117)				Smokers (*n* = 195)			
		Control (%)	Case (%)	OR (95% CI)	*P* value	Control (%)	Case (%)	OR (95% CI)	*p* value
miR-146a rs2910164	GG	29 (53.7%)	25 (39.7%)	1		52 (56%)	44 (43%)	1	
	GC	25 (46.3%)	33 (52.3%)	1.53 (0.72–3.22)	0.26	35 (38%)	48 (47%)	1.62 (0.89–2.93)	0.11
	CC	0 (0%)	5 (8.0%)	12.7 (0.67–241)	0.09	6 (6%)	10 (10%)	1.96 (0.66–5.85)	0.22

miR-155 rs767649	TT	42 (77.7%)	48 (76.1%)	1		60 (64.5%)	83 (81.3%)	1	
	TA	12 (22.3%)	12 (19.0%)	0.87 (0.35–2.15)	0.77	26 (27.9%)	16 (15.6%)	0.44 (0.21–0.90)	0.024
	AA	0 (0%)	3 (4.8%)	6.13 (0.30–122)	0.23	7 (7.5%)	3 (2.9%)	0.30 (0.07–1.24)	0.099

**Table 5:** Association of SNPs with NSCLC according to stage and subtypes.

Variable	rs2910164							rs767649						
	GG	CG	CC	Allele G frequency (%)	Allele C frequency (%)	Adjusted OR (95%CI)	*p* value	TT	TA	AA	Allele T frequency (%)	Allele A frequency (%)	Adjusted OR (95%CI)	*p* value
Stage I (*n* = 4)	2	2	0	6 (0.75)	2 (0.25)			4	0	0	4 (100)	0 (0%)
Stage II (*n* = 18)	7	10	1	24 (0.67)	12 (0.33)	0.66 (0.11–3.81)	0.64	15	3	0	33 (0.92)	3 (0.08)	1.06 (0.04–24.1)	0.96
Stage III (*n* = 33)	15	15	3	45 (0.68)	21 (0.32)	0.71 (0.13–3.84)	0.69	27	6	0	60 (0.91)	6 (0.09)	1.03 (0.04–21.4)	0.98
Stage IV (*n* = 110)	45	54	11	144 (0.65)	76 (0.35)	0.63 (0.12–3.20)	0.57	85	19	6	189 (0.86)	31 (0.14)	0.66 (0.03–12.7)	0.78
ADC type (*n* = 135)	57	65	13	179 (0.66)	91 (0.34)			106	25	4	237 (0.88)	33 (0.12)
SCC type (*n* = 24)	11	11	2	33 (0.69)	15 (0.31)	1.11 (0.57–2.16)	0.73	20	3	1	43 (0.89)	5 (0.11)	1.19 (0.44–3.23)	0.72
LCC type (*n* = 6)	1	5	0	7 (0.58)	5 (0.42)	0.71 (0.21–2.30)	0.75	5	0	1	10 (0.83)	2 (0.17)	0.69 (0.14–3.31)	0.64

**Table 6:** Association of NSCLC with alleles distribution (rs2910164G/C; rs767649T/A).

rs2910164	rs767649	Alleles	NSCLC (*n* = 165)	Control (*n* = 147)	OR (95% CI)	*p* value
G	T	GT	50% (146)	52% (135)	1 (reference)	
G	A	GA	11% (31)	16% (43)	0.71(0.30–1.69)	0.44
C	A	CA	7% (21)	7% (18)	1.04 (0.34–3.17)	0.94
C	T	CT	32% (92)	25% (64)	1.33 (0.69–2.55)	0.38

## Data Availability

The data used to support the findings of this study are available from the corresponding author upon request.

## Abbreviations

BRCA1: Breast cancer 1
miRNA: MicroRNA
Myc: Transcription factor C-MYC
NSCLC: Non-small-cell lung cancer
RFLP: Restriction fragment length polymorphism
PCR: Polymerase chain reaction
SNPs: Single nucleotide polymorphisms.
